# Scalp‐to‐cortex distance of left primary motor cortex and its computational head model: Implications for personalized neuromodulation

**DOI:** 10.1111/cns.13204

**Published:** 2019-08-16

**Authors:** Hanna Lu, Linda C. W. Lam, Yuping Ning

**Affiliations:** ^1^ Department of Psychiatry The Chinese University of Hong Kong Hong Kong China; ^2^ Guangdong Engineering Technology Research Center for Translational Medicine of Mental Disorders Guangzhou China; ^3^ The Affiliated Brain Hospital of Guangzhou Medical University Guangzhou China

**Keywords:** cortical thickness, dementia, electric field, morphometry, neuromodulation, primary motor cortex, scalp‐to‐cortex distance, simulation

## Abstract

**Background:**

Non‐invasive brain stimulation (NIBS) is increasingly used as a probe of function and therapeutics in experimental neuroscience and neurorehabilitation. Scalp‐to‐cortex distance (SCD), as a key parameter, has been shown to potentially impact on the electric field. This study aimed to examine the region‐specific SCD and its relationship with cognitive function in the context of age‐related brain atrophy.

**Methods:**

We analyzed the SCD and cortical thickness (CT) of left primary motor cortex (M1) in 164 cognitively normal (CN) adults and 43 dementia patients drawn from the Open Access Series of Imaging Studies (OASIS). The degree of brain atrophy was measured by the volume of ventricular system. Computational head model was developed to simulate the impact of SCD on the electric field.

**Results:**

Increased SCD of left M1 was only found in dementia patients (*P* < .001). When considering CT, the ratio of SCD to CT (*F* = 27.41, *P* < .001) showed better differential value than SCD. The SCD of left M1 was associated with worse global cognition (*r* = −.207, *P* = .011) and enlarged third ventricle (*r* = .241, *P* < .001). The electric field was consequently reduced with the increased SCD across cognitively normal elderly and dementia groups.

**Conclusions:**

Scalable distance measures, including SCD and CT, are markedly correlated with reduced electric field in dementia patients. The findings suggest that it is important to be aware of region‐specific distance measures when conducting NIBS‐based rehabilitation in individuals with brain atrophy.

## 1 INTRODUCTION

Noninvasive brain stimulation (NIBS), including transcranial magnetic stimulation (TMS) and transcranial direct current stimulation (tDCS), encompasses a broad array of treatments that target the disease‐specific regions or networks to achieve desired outcomes in neurorehabilitation.^1^, ^2^ It should be noted that TMS and transcranial eletrical stimulation (tES) use coil or electrode placed on the scalp to deliver a magnetic or electrical current through the scalp to the cortex where the stimulation levels are supposed to be attenuated with the distance.^3^, ^4^, ^5^ As highlighted in the NIBS guidelines,^6^ scalp‐to‐cortex distance (SCD) has been reported to critically influence the focality and strength of electric field induced by NIBS.^5^, ^7^

**Figure 1:**
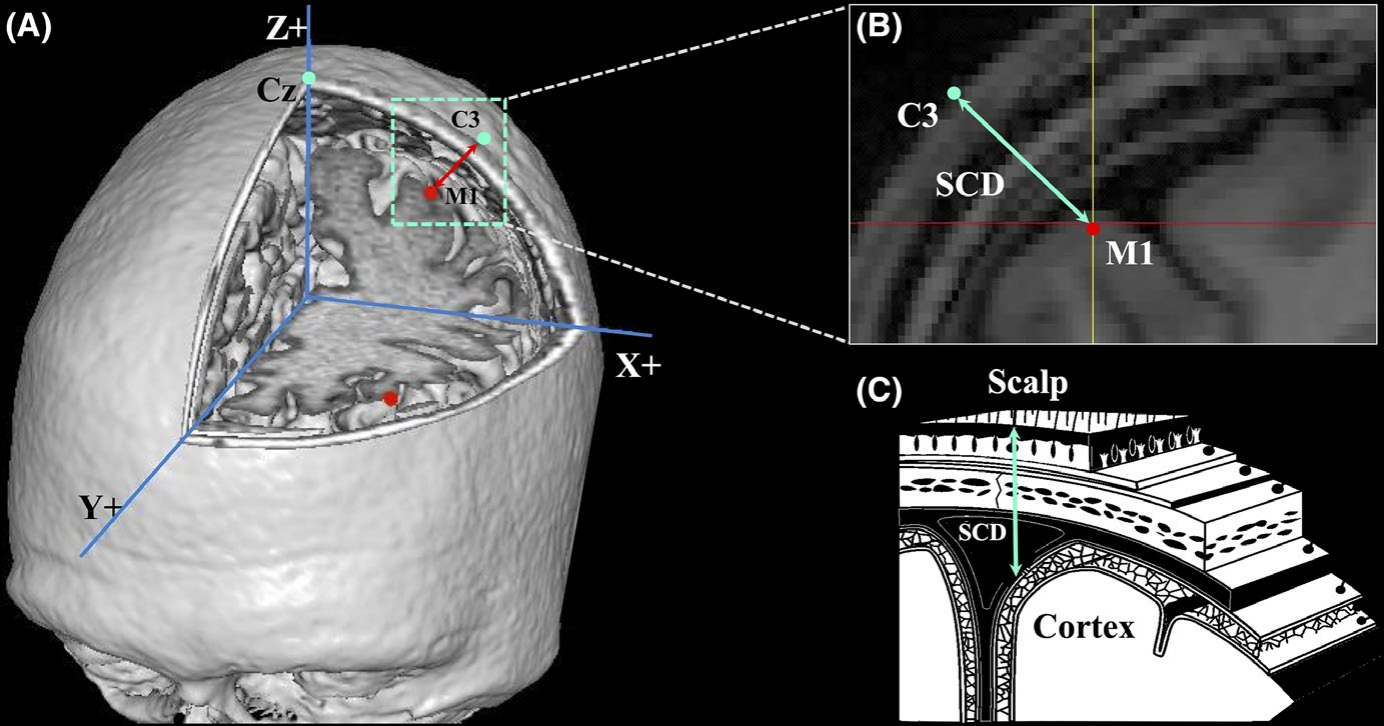
Illustration of three‐dimensional scalp‐to‐cortex distance (SCD), including (A) the stimulation target on the scalp and cortical surface, (B) distance measured from scalp to cortex, and (C) the anatomical layers of SCD

Basically, the SCD of left primary motor cortex (M1) is relatively higher than those of the other cortical regions.^8^ Prior evidence showed that the motor threshold (MT) measured at the scalp with single‐pulse TMS is highly dependent on the SCD of M1 (Figure 1).^9^, ^10^, ^11^ With regard to the increasing heterogeneity in adults with advancing age, the absence of the link between the SCD of M1 and cortical thickness raises the concerns about the stimulation power of stimulating other brain regions using the MT determined by stimulating left M1 in clinical populations, such as stroke and dementia,^12^ not even mentioned the seniors and dementia patients with cortical atrophy.

To contribute robust evidence to the scientific value and utility of SCD in brain stimulation and overcome some limitations of previous studies, we aimed to investigate the effects of age and dementia on the SCD and cortical thickness of left primary motor cortex (M1) in individuals with normal aging and dementia. Meanwhile, we also aimed to construct a computational head model to test the impact of SCD on the tDCS‐induced electric field.

## 2 METHODS

### 2.1 Participants

Two hundred and thirteen right‐handed participants across the adulthood (aged from 25‐85 years) were drawn from the Open Access Series of Imaging Studies (OASIS) (http://www.oasis-brains.org).^13^ Six cases were excluded due to the failures in the processing, including skull stripping (n = 2), segmentation (n = 1) and cortical reconstruction (n = 3). The remaining 207 cases were classified with regard to the chronological age and cognitive status, including young (aged from 25‐35 years), middle age (aged from 36‐60 years), old age (aged from 61‐85 years), and dementia patients. Age, gender, and available score of cognitive function for individuals in each of these groups were obtained directly from the OASIS dataset.

### 2.2 MRI data acquisition

As described by Marcus et al,^13^ structural magnetic resonance imaging (MRI) images of OASIS database were acquired on a 1.5 Tesla Vision scanner (Siemens, Erlangen, Germany) within a single session during which cushioning and thermoplastic face mask were employed to minimize head movement. T1‐weighted magnetization prepared rapid gradient echo (MPRAGE) sequence was empirically optimized for the gray‐white contrast, with repetition time (TR) = 9.7 ms, echo time (TE) = 4.0 ms, inversion time = 20 ms, delay time = 200 ms, flip angle = 10**°**, orientation = sagittal, resolution = 256 × 256 matrix, slices = 128, and thickness = 1.25 mm.

#### 2.2.1 Study 1 Surface‐based morphometry

**Figure 2:**
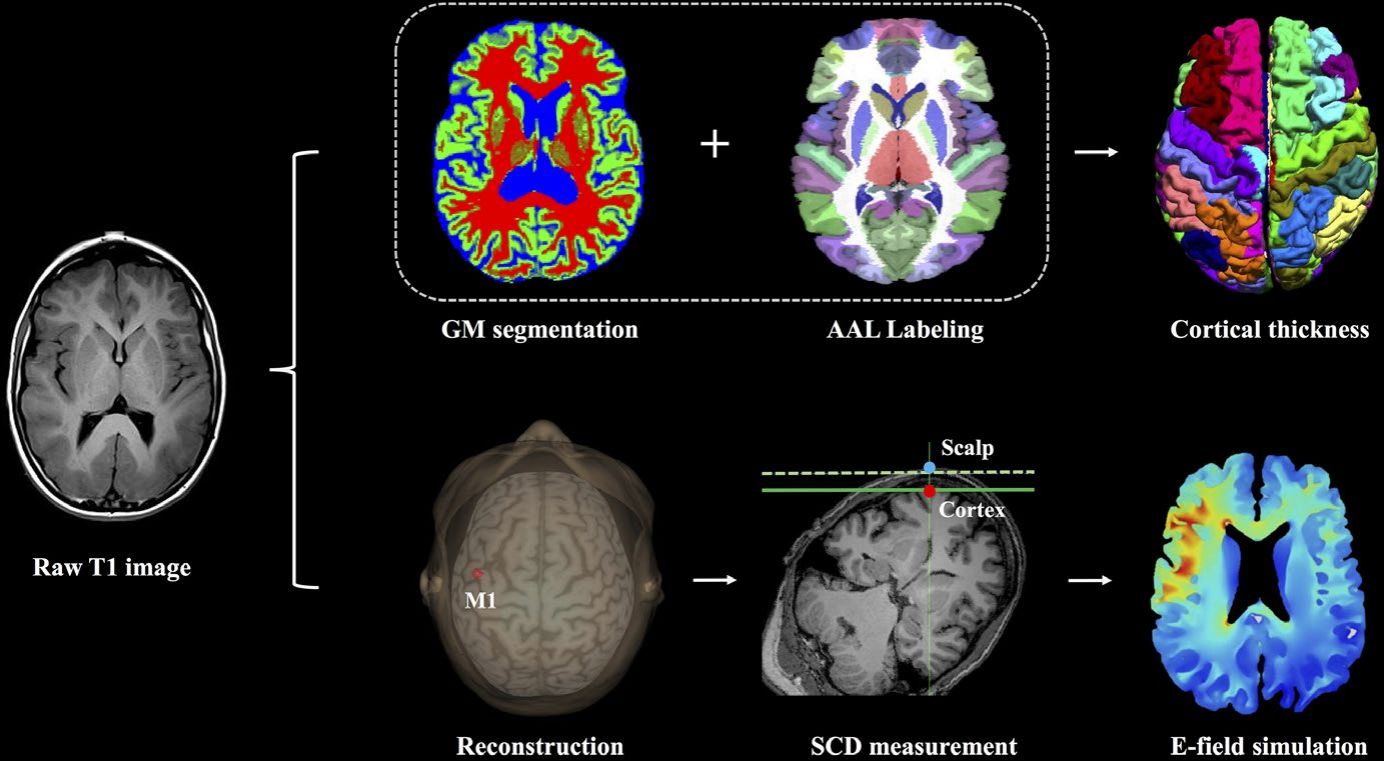
Analytic pipeline of scalp‐to‐cortex distance (SCD) and brain morphometry of left primary motor cortex (M1) and electric field (E‐field) simulation. Based on structural MRI data, surface‐based morphometry analysis was performed to calculate the cortical thickness of left M1 (ie, premotor gyrus in AAL template). In next step, MRI data were imported to neuronavigation system for reconstructing cerebral cortex, localizing the target, and measuring the SCD of left M1. Finally, SCD‐dependent E‐field simulation is conducted across age‐ and dementia‐specific groups

As shown in Figure 2, cortical thickness (CT) and cerebrospinal fluid (CSF) (ie, measure of brain atrophy) was analyzed by BrainSuite 14.0 (http://brainsuite.org/).^14^ BrainSuite is an automatic cortical surface identification integrated toolbox with the updated version of Brain Surface Extraction (BSE),^15^, ^16^ which is commonly used in aging studies.^17^, ^18^ We mapped the individual CT on the basis of Automated Anatomical Labeling (AAL) template by applying the following procedures: Firstly, we corrected the motion and removed the nonbrain voxels. Secondly, we segmented brain into gray matter, white matter, and cerebrospinal fluid. Thirdly, we coregistered individual MRI data to AAL template using a similarity transformation. Next, region‐specific CT was calculated as the smallest distance (in millimeters) of each point on the external surface of gray matter from the outermost surface of the white matter. At each step, we visually checked the outputs and manually corrected when there are segmentation errors (ie, nonbrain tissue).

#### 2.2.2 Study 2 A reconstructive approach to measure SCD

##### Reconstruction system

Brainsight neuronavigation system (Rogue Resolutions Ltd) was employed to integrate structural MRI images to perform automatically three‐dimensional (3D) reconstruction of head and cerebral cortex. For each subject, we firstly performed an MRI‐to‐head coregistration, and then identified and adjusted the coregistered brain by the AC‐PC line into Montreal Neurological Institute (MNI) space. After normalization, the location of predefined target was labeled with the coordinates in MNI space (*x, y, z*) (Figure 2).

##### Localizing left M1 in 3D space

Based on the cortical surface reconstruction, we identified the locations of left M1 individually. In NIBS practice, the hand representation within left M1 was determined according to the anatomical criteria with the MNI coordinates as [x = −42, y = −16, z = 68],^19^ representing as “hook sign” on sagittal plane. The location of left M1 was verified within the gray matter on the top of paracentral gyrus (ie, Brodmann area 4, BA4).

##### Measurement of SCD

To better mimic the stimulation, the corresponding location of left M1 on the scalp is conducted in Brainsight neuronavigation system with pointing back the cursor to the scalp and adjusted with the orientation of the coil from the midline at 45°. The target location and the angle with coil were checked visually and individually. The accuracy of the target was further verified in the planes of axial, coronal, and sagittal. The SCD of left M1 was measured in the neuronavigation system as the distance from the scalp to cortex (Figure 1). Euclidean distance (*D_i_*), as a geometric index, is used to measure the distance between two points locating on the scalp (*x_s_, y_s_, z_s_*) and on the cortex (*x_c_, y_c_, z_c_*) with the formula 20:

$$D_{i}=\sqrt{{\left(x{}_{s}-x_{c}\right)}^{2}+{\left(y_{s}-y_{c}\right)}^{2}+{\left(z_{s}-z_{c}\right)}^{2}}.$$

#### 2.2.3 Study 3 Simulating the effect of SCD on electric field

To examine the impact of SCD on electric field (E‐field), a head model was constructed based on structural MRI images (1 mm^3^ isotropic resolution) by SPHERES. The SPHERES 1.0 is a stand‐alone graphical user interface application that allows the considerations of arbitrary montages and adjustment of brain parameters on a concentric sphere model by leveraging an analytical solution (http://www.parralab.org/spheres/).^21^, ^22^ The surface meshes were obtained from the T1 data adopted from BrainSuite. Next, we used SPHERES to simulate the effect of SCD on electric field in a scenario of anodal tDCS over left M1.

### 2.3 Statistical analysis

Group differences of demographics and cognitive function were tested either with chi‐square (*χ* ^2^) test for categorical variable or with ANOVA for continuous variables. CT and the volume of ventricular system were calculated individually. The comparisons of CT and the volume of ventricular system between groups were conducted by the code embedded in MATLAB R2016a (http://neuroimage.usc.edu/neuro/Resources/BST_SVReg_Utilities). Multiple comparison correction was used by the above code using false discovery rate (FDR) estimation.^23^ Pearson correlation coefficient was used to detect the relationship between age, cognitive score, cortical thickness, and distance measures. Bonferroni correction was addressed to reduce the chances of obtaining false‐positive results of correlation analysis.^24^, ^25^ The chi‐square test, one‐way ANOVA, and Pearson correlation analysis were performed by IBM SPSS Statistics (Version 20).

## 3 RESULTS

### 3.1 Study 1 Morphometric features of left M1

Of the 207 participants, 164 were defined as cognitively normal (CN) adults and 43 defined as dementia patients. Based on the definition of World Health Organization (WHO),^26^ 164 CN adults were divided into three age‐specific groups, including young (aged from 25‐35 years), middle age (aged from 36‐59 years), and old age (aged from 60‐85 years). As Table 1 shown, gender ratio across four groups was similar (*F* = 2.35, *P* = .078). The scores of Mini‐Mental State Examination (MMSE) between middle age and old age groups were comparable (*F* = 1.45, *P* = .15). Compared with CN adults, dementia patients showed significant declined global cognitive efficiency (MMSE: CN adults: 29.47 ± 0.75, dementia patients: 20.21 ± 3.61, *t* = 14.69, *P* < .001).

**Table 1:** Demographics, cognitive function, and morphometric features across groups

	Young (n = 40)	Middle age (n = 62)	Old age (n = 62)	Dementia (n = 43)	*F* (*χ* ^2^)	*P* value
Age (years)	29.43 ± 3.97	50.16 ± 5.35	75.73 ± 8.09	75.95 ± 6.82	658.86	<.001
Gender (M:F)	24:16	42:20	42:20	25:18	2.35	.078
MMSE score	‐	29.47 ± 0.75	28.03 ± 2.52	20.21 ± 3.61	166.69	<.001
TIV (×10^3^ mm^3^)	1670.1 ± 302.7	1665.9 ± 105.1	1664.5 ± 181.8	1358.8 ± 200.1	165.81	<.001

Note

Data are raw scores and presented as mean ± SD.

Abbreviations: MMSE, Mini‐Mental State Examination; TIV, total intracranial volume.

**Figure 3:**
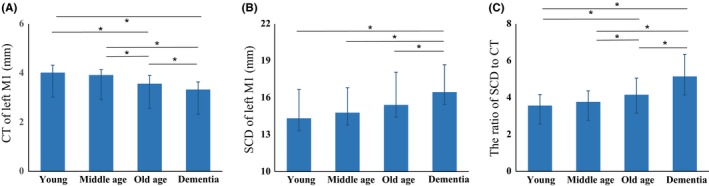
Comparisons of cortical thickness (CT) and SCD of left primary motor cortex (M1) across groups. A, Group‐wise differences of CT. B, Group‐wise differences of three‐dimensional SCD. C, CT‐adjusted SCD of left M1, rather than SCD presents better discriminative utility than SCD across age‐ and dementia‐specific groups

Within CN adults, group‐wise differences were found in the CT of left M1 (*F* = 24.18, *P* < .001), of which the cases with old age had thinner CT of left M1 than the ones with young and middle age. Dementia patients showed significant cortical reductions in left M1 (*F* = 39.68, *P* < .001, Figure 3A). Using gender and global CT as covariates, age was negatively correlated with the CT of left M1 (*r* = −.539, *P* < .001) (Table 2).

**Table 2:** Scalp‐to‐cortex distance and cortical thickness of left M1 across groups

Morphometric measures	Young (n = 40)	Middle age (n = 62)	Old age (n = 55)	Dementia (n = 43)	*F* (*χ* ^2^)	*P* value
CT (mm)	4.02 ± 0.26	3.93 ± 0.23	3.65 ± 0.33	3.33 ± 0.49	39.68	<.001
SCD (mm)	14.33 ± 2.49	14.79 ± 2.62	15.42 ± 2.81	16.93 ± 4.21	6.28	<.001
SCD/CT	3.57 ± 0.66	3.77 ± 0.68	4.25 ± 0.89	5.15 ± 1.25	27.41	<.001

Note

Data are raw scores and presented as mean ± SD.

Abbreviations: CT, cortical thickness; M1, primary motor area; SCD, scalp‐to‐cortex distance.

### 3.2 Study 2 Geometric SCD of left M1

The SCD of left M1 as calculated based on MNI coordinates on the scalp (*x_s_, y_s_, z_s_*) and the ones on cerebral cortex (*x_c_, y_c_, z_c_*) identified the precise location in neuronavigation system visually (Figure 2). Prominent difference of the SCD of left M1 was found across the groups (*F* = 6.28, *P* < .001, Figure 3B). When considering CT, the ratio of SCD to CT (*F* = 27.41, *P* < .001) showed better differential value than SCD (Figure 3C).

Using gender and global CT as covariates, age was modestly correlated with the SCD of left M1 (*r* = .142, *P* = .037). The SCD of left M1 was associated with worse MMSE score (*r* = −.207, *P* = .011). Enlarged third ventricle was prominently correlated with the SCD of left M1 (*r* = .241, *P* < .001) and the ratio of SCD to CT of left M1 (*r* = .282, *P* < .001).

### 3.3 Study 3 Simulation of SCD‐dependent electric field

**Figure 4:**
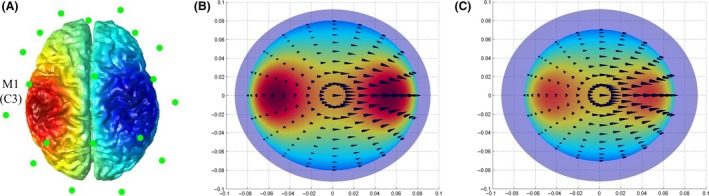
Computational head model of anode tDCS over left primary motor cortex (M1) with a 5 × 5 cm electrode, depicting the cortical surface with EEG electrodes (A) and the distribution of the electric field's magnitude in individuals with old age (B) and dementia (with comparable age) (C)

To create a computational head model, structural MRI images were registered and normalized to MNI space, and then the cortical surface was reconstructed based on the processed images from BrainSuite. Head model was prepared for tDCS‐induced E‐field simulation by adding a 5 × 5 cm (25 cm^2^) rectangular anode electrode on the scalp centered over left M1 and adding cathodal electrode over right M1 (Figure 4A) (ie, C3 position of the 10‐20 international EEG system).

Using the SCD of left M1 as the key parameter in SPHERES, the isotropic conductivity of the tissues in this head model were adopted with default setting, including: 0.3 S/m for the scalp, 0.03 S/m for the skull, 2 S/m for the CSF, and 0.03 S/m for the cortical tissue.^27^, ^28^, ^29^ Compared with age‐matched CN adults (Figure 4B), the spatial distributions of the anode tDCS‐induced E‐field were prominently decreased in dementia group (Figure 4C).

## 4 DISCUSSION

In this study, we targeted and measured the scalp‐to‐cortex distance of left primary motor cortex (M1) in the stereotaxic neuronavigation system. Further, we tested the SCD‐dependent impact on the tDCS‐induced E‐field in computational head model. To the best of our knowledge, this study showed for the first time that the age‐ and dementia‐related SCD of left M1 and its impact on the tDCS‐induced E‐field by combining surface‐based morphometry, cortical reconstruction, and simulation.

Reductions in brain morphometry and enlarged ventricular system with advancing age and dementia are well documented.^30^, ^31^ However, the critical question pinpointed here is whether the measures with the same scale (ie, mm) reflect the comparable morphometric features in geometric space. Indeed, it is evident that we can combine CT with preintervention parameter setting (ie, SCD) and further assist the personalized intervention. Meanwhile, the associations between SCD and CT in late adulthood also highlight the rational for taking CT into account when measuring SCD. Cerebral cortex contains six layers of neuronal cells with specific cytoarchitecture and neuromodulators,^32^ of which the thickness of the six layers is computed as CT in surface‐based morphometry analysis. The ultimate aim of NIBS is to precisely stimulate the interneurons in specific layers,^33^ thus measuring SCD alone is insufficient in individuals with brain atrophy. In addition to SCD adjustment, we proposed using a personalized factor (ie, ratio of SCD to CT) to optimize the TMS dose for the treatment procedure in age‐ and disease‐dependent populations.

It is notable that, beyond a technological parameter, SCD should be considered as a measure of brain morphometry. Except for the same scale (ie, mm) SCD and CT had, the vector‐like properties of SCD, including: (a) direction: SCD is a line starting from the point on the scalp and ending at the point on cerebral cortex and (b) magnitude: the measure of SCD representing the length of the line, also have profound impact on the modeling of tDCS. For instance, recent evidence confirmed that the directionality (or orientation) of the current injection can critically influence the field potential of the targeted region during transcranial electrical stimulation.^34^, ^35^ Therefore, region‐specific SCD not only reflects the individual morphometric features, but also provides a useful and dynamic parameter to optimize the therapeutic protocol.

Another intriguing part is that the link between SCD and the volume of ventricular system indicates the region‐specific and SCD‐related ventricular enlargement and highlights the possible way to make good use of the enlarged ventricular system in NIBS. As mentioned in electric field simulation, CSF has the highest conductivity in brain, which is 66.7 times higher than cerebral cortex. In this regard, whether the increased CSF (ie, enlarged ventricle), as super‐conductor, is sufficient to cover the reduced E‐field due to increased SCD, and to what extent it interacts with the energy delivered into the brain will be the questions that may be of interest in simulation neuroscience.

### 4.1 Limitations and future directions

The findings of this study should be interpreted with caution due to the limitations: (a) Several key variables, including years of education, genetic factors, domain‐specific cognitive function, and medical history were not provided in this dataset; (b) the measure of SCD was based on a “point‐to‐point” approach; (c) the T1 MRI images were draw from cross‐sectional data, which have limited interpretation on aging effect on SCD.

A way forward in the application of NIBS is to tailor the intervention to individual patients or subgroups of patients based on chronological or morphometric profiles. Furthermore, the large variability in the therapeutic effect of NIBS studies may be caused by the inter‐individual and intra‐individual region‐specific SCD and morphometric features. Combing anatomical changes and atrophy in the targeted brain region shows a possible way forward to improve future NIBS practice.

## 5 CONCLUSION

Collectively, in contrast to normal aging, the SCD of left M1 in dementia patients is prominently increased and accompanied by reduced E‐field. We recommend for developing a thoughtful process of disclosure for personalized NIBS strategies. Beyond SCD‐adjusted MT,^36^, ^37^ we can optimize the stimulation parameter based on age‐ and dementia‐specific morphometric profiles. A prominent linkage between SCD and tDCS‐induced E‐field also illustrates the necessity of integrating neuroimaging data to NIBS in the future.

## CONFLICT OF INTEREST

The authors declare that they have no competing interests, and there are no financial competing interests to declare in relation to this manuscript.

## ETHICAL APPROVAL

For the purposes of this study, we used the structural images of the OASIS that was previously collected under several study protocols at Washington University. This study was carried out in accordance with the recommendations of the University's Institutional Review Board (IRB). The protocol was approved by the University's IRB. All participants gave written informed consent in accordance with the Declaration of Helsinki. The University's IRB also provided explicit approval for open sharing of the anonymized data.

## CONSENT FOR PUBLICATION

All authors have approved the manuscript for submission. All participants gave written informed consent to publish these data. No individual details, images, or videos are included in the manuscript.

## Data Availability

The dataset supporting the conclusions of this article can be found at the official website of the OASIS project: www.oasis-brains.org and LANDSCAPE project: www.thebrainx.com/landscape.
